# Comment on “Which fraction of stone wool fibre surface remains uncoated by binder? A detailed analysis by time-of-flight secondary ion mass spectrometry and X-ray photoelectron spectroscopy” by Hirth *et al.*, 2021, *RSC Adv.*, **11**, 39545, DOI: 10.1039/d1ra06251d

**DOI:** 10.1039/d2ra07959c

**Published:** 2023-06-02

**Authors:** Denis V. Okhrimenko, Marcel Ceccato, Sven Tougaard, Morten Foss, Eric Pezennec, Mette Solvang

**Affiliations:** a ROCKWOOL A/S Denmark; b Interdisciplinary Nanoscience Center (iNANO), Aarhus University Denmark; c University of Southern Denmark Denmark; d Knauf Insulation Belgium

## Abstract

The article mentioned in the title of this comment paper reports on an investigation of the organic binder presence and distribution on stone wool fibres with surface sensitive techniques (X-ray photoelectron spectroscopy (XPS), QUASES XPS modelling, time-of-flight secondary ion mass spectrometry (ToF-SIMS) mapping) and attempts to correlate the results with fibre performance in *in vitro* acellular biosolubility tests. However, the study has assumptions, hypothesis and results that do not take into account the recognised science and regulations on biopersistence of stone wool fibres, limitations of the utilized surface sensitive techniques and modelling approach and it contains a contradiction with biosolubility experiments. In this comment article, we discuss these points, propose improved QUASES XPS modelling and present recent ToF-SIMS mapping results that reflect biosolubility behaviour of the stone wool fibres.

## Abstract 2

A thin organic layer (binder, oil, adventitious carbon) on a stone wool fibre surface is incomplete and inhomogeneous and leaves the bare fibre surface available for dissolution in lung fluid.

## Graphical abstract

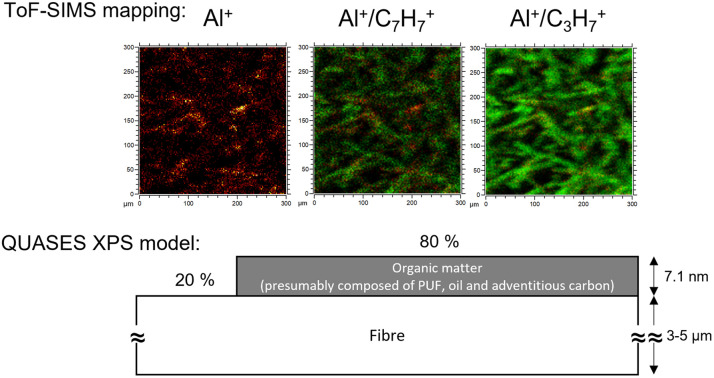

## Introduction

Hirth and colleagues^1^ have recently investigated the distribution of organic material (binder and mineral oil) on stone wool fibres. The work follows up previous publications by the authors: Wohlleben *et al.*, 2017 ^2^ and Sauer *et al.*, 2021.^3^ The starting point for their publications is the authors' view that hazard assessment of man-made vitreous fibres (MMVF) is solely based on biodurability measurements of naked fibres (*i.e.* without binder). Similar to the previous discussion,^4^ we would like to bring attention to the fact that *in vitro* acellular biodurability tests either on fibres with or without binder are not relevant for the hazard assessment and regulations on mineral wool fibres. Actually, MMVF hazard assessment includes investigation of fibre biopersistence *via in vivo* animal studies with typically nasal inhalation or intratracheal installations of fibres produced without binder^5,6^ (Note Q of the European Regulation (EC) No. 1272/2008 (CLP) (EC2008)) and epidemiological studies on workers, where the impact of fibres produced with binder is studied, both recognised at international and European level.^7–13^ Despite this, the papers^1–3^ attempted to find differences in the *in vitro* acellular behaviour of fibres with and without binder, using binder removal techniques that modify fibre chemistry,^14,15^ wettability and thus likely solubility.^16,17^ The paper^1^ explored the distribution of binder (presumably phenol-urea-formaldehyde, PUF) on stone wool fibres and tried to find a correlation between dissolution rate of stone wool measured in a simulated lung fluid (phagolysosomal simulant fluid, PSF) and the amount and thickness of organic material on the fibre surface. The article^1^ reports the use of surface sensitive techniques, such as X-ray photoelectron spectroscopy (XPS), time-of-flight secondary ion mass spectroscopy (ToF-SIMS) mapping and modelled XPS data with QUASES software.^18^ The general findings of the paper about organic matter obtained with XPS and QUASES XPS modelling are in line with previously published results on stone wool samples.^19,20^ However, we would like to stress several points concerning the assumptions and hypotheses in the publication,^1^ analytical techniques limitations in spatial resolutions and interpretation of results and as well present the newest results using QUASES XPS and ToF-SIMS on stone wool samples with PUF binder.

## Discussion

### The assumptions on biopersistence assessment

Hirth *et al.*^1^ state that fibre biodurability is currently assessed on “naked” fibres (*i.e.* produced without binder) because there is an assumption that fibres produced with organic matter (binder) would not have a completely coating of the fibre, and that this would rather be localised in the areas where fibres enter into contact and thus leaving large fraction of the fibre surface uncoated. It has to be mentioned that for biopersistence tests, fibres produced without binder are traditionally used also for other reasons. In *in vivo* studies,^5,6^ fibres without binder are recommended because aerosol sizing, fibre diameter measurements and sterilization of the test material are impaired by the presence of binder.††Personal Communication 2019, F. Schulz, Fraunhofer Institute for Toxicology and Experimental Medicine ITEM Hannover, Germany. Binder presence also causes fibres to agglomerate, which may result in suffocation of the animals after intratracheal instillation. As in real inhalation scenario, respirable fibres are present as single fibres, while larger agglomerates are not able to reach the alveolar region of the lung, this should also be avoided in the *in vivo* tests by using fibres without binder.† However, in an earlier *in vivo* study it is shown that stone wool fibres produced with and without binder perform similarly (Wagner *et al.*, 1982,^21^ Experiment 1 for stone wool fibres injected intrapleurally). Fibre safety is also largely explored by epidemiological studies^7–13^ at manufacturing sites, where no adverse effects of stone wool fibres as produced, meaning possibly with binder,^31^ are found on workers. Epidemiology is the first type of studies that IARC^22^ is using to investigate carcinogenicity of substances, including stone wool fibres, followed by *in vivo* investigations. Thus, today, fibres' biosolubility *in vitro* (acellular and cellular) is not the key indicator to assess the stone wool fibres hazard assessment.

### Incomplete information on composition of test material and organic matter

No details are provided in the paper regarding composition of the stone wool fibres, unlike in previous publications by the authors.^2,3^ The lack of information about fibre composition makes it difficult to follow the dependence of the fibre dissolution rate on the inorganic composition of the fibres, which authors concluded to be the main factor.

The authors^1^ state that they expect phenolic resin to be commonly used as a binder based on their finding of traces of nitrogen but no further information on the organic binder chemical composition is provided. In the paper the binder appears to be treated as a classic organic molecule without further differentiation of other binder components (such as oil, coupling agent *etc.*). We further note that providing SEM images (a standard technique for the study of microstructure on fibre surfaces) of the stone wool fibres would have been beneficial and would have enabled the distinction between micrometre size areas with binder and the rest of the surface.

### Resolution of ToF-SIMS and XPS results

**Fig. 1:**
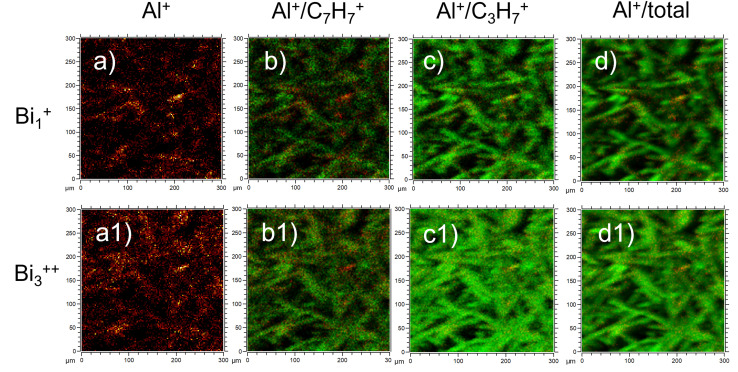
ToF-SIMS imaging results of F3 stone wool fibres (3.6 wt% PUF binder, 0.1 wt% mineral oil) from Barly *et al.*, 2019:^23^ (a-a1) Al^+^; (b-b1) overlay of Al^+^ (red) and C_7_H_7_^+^ (green); (c-c1) overlay of Al^+^ (red) and C_3_H_7_^+^ (green) and (d-d1) overlay Al^+^ (red) and total intensity (green). The images (a–d) are obtained with Bi_1_^+^ ion source and other settings similar to those in Okhrimenko *et al.*, 2022.^16^ The images (a1–d1) are obtained with Bi_3_^++^ ion source similar to Hirth *et al.*, 2021.^1^

We would like to highlight that the used low ToF-SIMS mapping resolution could give the impression that the signal coming from the fibre surface is dominated by carbon from oil and binder (as the oil and binder are on the top of the fibres). We do not think that this is sufficient documentation in the paper to conclude, that fibres are coated almost at 100%. In another recent study,^16^ yet with different ToF-SIMS resolution (300 × 300 μm; 128 × 128 pixels), ion source (Bi_1_^+^) and binder applied to the fibres (sugar-based binder, SBB), it was possible to observe a signal from the fibre substrate itself (Al^+^), indicating that binder does not completely coat the fibre surface. In Fig. 1 we present recent ToF-SIMS imaging results on stone wool fibres with PUF binder from Barly *et al.*, 2019 ^23^ (F3 sample, 3.6 wt% PUF binder, 0.1 wt% mineral oil) performed with the same ToF-SIMS settings as in Okhrimenko *et al.*, 2022.^16^ The signal from fibre substrate (Al^+^, Fig. 1a, a1) is dominating over the signal from organic layer originated likely from PUF binder (C_7_H_7_^+^) and oil (C_3_H_7_^+^) in many areas on the fibre surface (Fig. 1b, b1, c and c1), indicating that PUF binder and mineral oil coverage is neither uniform nor complete.

It is important to consider the influence of the ToF-SIMS mapping spatial resolution and other settings on the conclusions about binder distribution on the fibres. This would help to understand why fibres with different binders (PUF and SBB) and fibres without any binder perform similar in dissolution tests as found in.^16,23^ We also note that the journal number and year for the work by Barly *et al.*, 2019 ^23^ in the Notes and references of Hirth *et al.*, 2021 ^1^ are not correct.

For the XPS results, no survey (wide-scan) spectra of the studied stone wool samples are presented. This does not allow to check the presence of additional chemical elements in the different stone wool samples that were compared. Complete survey spectra would have enabled the authors and the readers to get a first qualitative view of comparison between the samples. Moreover, it would be beneficial for the readers if it was acknowledged that both methods, XPS and ToF-SIMS, are extremely sensitive towards contaminations by adventitious carbon, which can originate from fibre storage and handling, as well as from apparatus *in situ*, and interfere with the performed analysis, reducing its representativity.

### Limitations of the modelling of surface layer thickness

We would like to note that the results of the QUASES XPS modelling to support the hypothesis that binder and mineral oil completely shield the surface of the fibres should be interpreted with greatest caution.

QUASES XPS modelling works the best if reference spectra are available, *i.e.* in this case this would be a spectrum of “naked” fibres without organic matter on their surface. In the absence of reference spectra, several models describing experimental XPS spectra are possible. The authors^1^ chose to simulate the surface layer in a similar way as in the study by Okhrimenko *et al.*, 2018,^20^*i.e.* as a uniform carbon layer with thickness 1–3 nm on top of the fibres. While the approach of using the background of the Si and Al XPS peaks to determine the binder distribution can be relevant at the considered thicknesses (≤10 nm), we note that binder droplets can be thicker (30–50 nm) and they are “blind” spots for QUASES analysis.

**Fig. 2:**
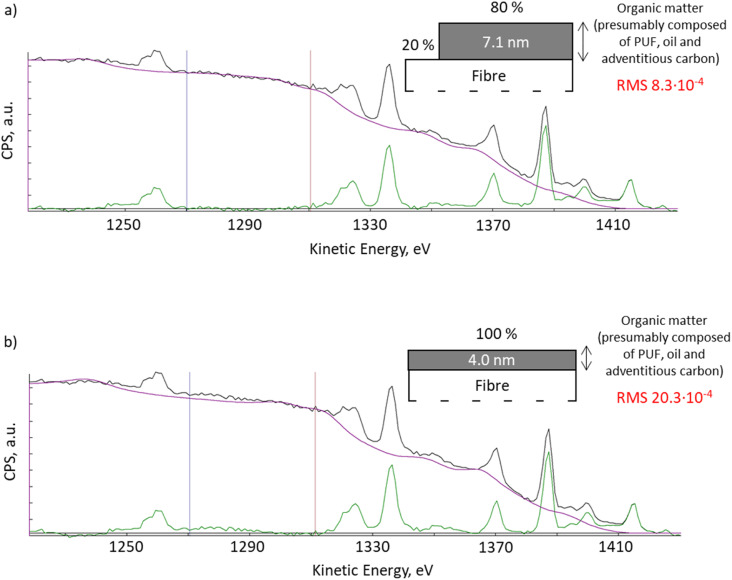
XPS spectra QUASES fitting using model: (a) with 80% of the surface covered with 7.1 nm organic layer and 20% of the uncoated surface; (b) uniform organic layer of 4.0 nm thick (Okhrimenko *et al.*, 2018).^20^

With QUASES software version 7.5, the results from Okhrimenko *et al.*, 2018 ^20^ can be re-evaluated using the automation option. QUASES v.7.5 uses the simplex method to determine the combination of all structure parameters which gives the minimum root mean square, RMS, between the spectrum and the background in the desired energy range. Using the automated structure determination facility that varies the structure until the RMS deviation in the 1270 to 1310 eV energy range reaches a minimum, we observed an improved fit of the XPS spectra when the software applies the model where 20% of the fibre surface remained uncoated (Fig. 2a, RMS 8.3 × 10^−4^), compared to the previous fitting with a 4 nm thick uniform layer presented in Okhrimenko *et al.*, 2018 ^20^ (Fig. 2b, RMS 20.3 × 10^−4^). This reduction in RMS is substantial but it should also be supplemented by a visual inspection of the spectra: the fit in Fig. 2a is seen to be virtually perfect in the full energy range from 1270 to 1295 eV, whereas there are clear deviations in Fig. 2b in this energy range. Any other structural model also gives substantially worse fits to the background.

Presence of the organics-free areas correlates with ToF-SIMS mapping results presented in our work for PUF treated fibres and by Okhrimenko *et al.*, 2022 ^16^ for SBB treated fibres and also with the similar dissolution behaviour of the fibre material irrespective if binder was applied or not^23^ and which type of the binder was applied.^16^ To sum up, the realization in the paper^1^ analyses without reference to neither the substrate nor the binders and without proof of the goodness of the modelling (only one example provided with narrow energy range, 1225–1345 eV) and the fact that their XPS spectrum was recorded with a rather low signal to noise level does not allow to ensure the solidity of the results and the findings. In addition, we note that information about how the general background is accounted for in the analysed energy region would have been very helpful. In QUASES XPS this is done by subtracting a straight which is fitted to the spectrum on the high energy side of the region. However, if peaks are present in this region (which is clearly the case here as seen in Fig. 2), the slope of this line can be uncertain and this adds to the uncertainty of the analysis. We avoid this problem by including in the analysis all peaks on the high energy side in the full energy range.

### Fibre dissolution and dissolution rate evaluation

The paper^1^ acknowledges that binder thickness is not a predictor for dissolution rates. This is confirmed by Fig. 7a in the paper,^1^ showing no correlation between dissolution rates and total binder content determined with thermogravimetry (TGA). It even shows that there is a reverse dependence of the dissolution rate on organic layer thickness in Fig. 7b of the paper^1^ (*i.e.* the higher the thickness of the organic layer, the faster fibres dissolve). The results contradict with the authors'^1^ hypothesis that the fibres are completely coated with the binder. The same authors previously demonstrated that mass loss of fibre with binder can reach up to 10% after 30 days of dissolution in PSF^2^ and higher in liquids with citrate (up to 100% within few days^3^). Taking into account such mass losses during dissolution and no time delay of the dissolution in the beginning of the tests, one can hardly expect any surface shielding effects by binder/organic layer shortly after beginning the dissolution test.

The observed fibre (with binder) dissolution can be explained by the fact that the organic layer on the fibre surface is incomplete and inhomogeneous in reality and leaves bare surface available for dissolution, as we have just shown with the newest QUASES XPS modelling and ToF-SIMS results presented here for PUF- and recently for SBB-treated fibers.^16^

Therefore, Hirth *et al.*, 2021 ^1^ results confirmed that binder presence cannot affect the dissolution of the stone wool fibres. It was shown that it is the inorganic chemical composition of the fibre that is among of the prime factors in *in vivo* pathogenicity^5,6^ and for *in vitro* cellular^24,25^ and acellular^26–28^ dissolution rates. Besides that, experimental conditions^16,28–30^ (*e.g.* fluid flow rate to sample surface area ratio, fluid composition, temperature, pH, dynamic or batch experiment and sample preparation) are crucial for the determination of the dissolution rates in *in vitro* acellular studies.

## Conclusions

In conclusion, we find that there are several methodological limitations in the article, which might provide an incorrect image of the dissolution and biosolubility of stone wool fibres. The conclusions made by Hirth *et al.*, 2021 ^1^ are in contrast with the existing science and regulations on biopersistence of stone wool fibres and other MMVF fibres. The present authors hope that provided comments, the additional examples of QUASES XPS modelling approach and application of ToF-SIMS mapping technique would support a better understanding of the biosolubility of the stone wool fibres, accepted terminology and existing regulations on MMVF biopersistence.

## Conflicts of interest

The authors declare following competing financial interest(s): D. V. O. and M. S. are employees of ROCKWOOL A/S, a company producing stone wool fibres. E. P. is employed by Knauf Insulation, a company producing stone and glass wool fibres.

## Supplementary Material
